# An Off-Target Nucleostemin RNAi Inhibits Growth in Human Glioblastoma-Derived Cancer Stem Cells

**DOI:** 10.1371/journal.pone.0028753

**Published:** 2011-12-12

**Authors:** Jon Gil-Ranedo, Marina Mendiburu-Eliçabe, Mercedes García-Villanueva, Diego Medina, Marta del Álamo, Marta Izquierdo

**Affiliations:** 1 Departamento de Biología Molecular, Universidad Autónoma de Madrid, Centro de Biología Molecular Severo Ochoa, Consejo Superior de Investigaciones Científicas, Instituto Sanitario Ramón y Cajal, Madrid, Spain; 2 Servicio de Anatomía Patológica, Hospital Universitario Ramón y Cajal, Madrid, Spain; 3 Servicio de Neurocirugía, Hospital Universitario Ramón y Cajal, Instituto Sanitario Ramón y Cajal, Madrid, Spain; Cedars-Sinai Medical Center, United States of America

## Abstract

Glioblastomas (GBM) may contain a variable proportion of active cancer stem cells (CSCs) capable of self-renewal, of aggregating into CD133^+^ neurospheres, and to develop intracranial tumors that phenocopy the original ones. We hypothesized that nucleostemin may contribute to cancer stem cell biology as these cells share characteristics with normal stem cells. Here we report that nucleostemin is expressed in GBM-CSCs isolated from patient samples, and that its expression, conversely to what it has been described for ordinary stem cells, does not disappear when cells are differentiated. The significance of nucleostemin expression in CSCs was addressed by targeting the corresponding mRNA using lentivirally transduced short hairpin RNA (shRNA). In doing so, we found an off-target nucleostemin RNAi (shRNA22) that abolishes proliferation and induces apoptosis in GBM-CSCs. Furthermore, in the presence of shRNA22, GBM-CSCs failed to form neurospheres *in vitro* or grow on soft agar. When these cells are xenotransplanted into the brains of nude rats, tumor development is significantly delayed. Attempts were made to identify the primary target/s of shRNA22, suggesting a transcription factor involved in one of the MAP-kinases signaling-pathways or multiple targets. The use of this shRNA may contribute to develop new therapeutic approaches for this incurable type of brain tumor.

## Introduction

Glioblastoma multiforme is one of the most malignant and common of all astrocytic tumors [1]. The growth pattern of GBM is highly infiltrative, rendering a surgical cure very difficult and resulting in very poor survival outcomes that have improved only marginally in the past several decades [2]. The cancer stem cell hypothesis suggests that tumors are organized in a hierarchy with a subpopulation of CSCs responsible for tumor progression, maintenance, and recurrence [3]. Cells with stem-like properties were initially identified in acute myeloid leukaemia [4], and at present their existence has been confirmed in breast cancer [5], medulloblastoma and glioblastoma [6], prostate cancer [7], melanoma [8], ovarian cancer [9], head and neck squamous carcinomas [10], colon cancer [11], pancreatic cancer [12] and lung cancer [13], among others. In glioblastoma, relapses normally follow treatment, probably because CSCs are highly infiltrative, selectively resistant to radiotherapies, chemotherapies [14], [15], [16], immunotherapies [17], and promote angiogenic activity. Moreover, chemo- and radio- therapies may prime brain tumor CSCs to enhance their stem-cell-like characteristics [18]. This population of CSCs is highly tumorigenic and phenocopy the original tumor in rodent xenograft models [19], [20]. Approaches to force CSCs to differentiate to cells with limited, or no cell division attributes, by exposing them to bone morphogenetic proteins for example, were used to render them more vulnerable to conventional therapies, and showed considerable efficacy in mouse models [21]. Understanding the basic biology of cancer stem cells is a key feature before moving into putative treatments to eliminate them.

Nucleostemin is a GTP-binding protein, so called because of its nucleolar localization and preferential expression in stem cells [22]. Although the protein is predominantly present in embryonic and adult stem cells, it is also expressed in several transformed cell lines and tumors [23], [24]. Nucleostemin, on the other hand, is abruptly down-regulated during differentiation prior to terminal cell division. This protein was first identified in adult rat neural stem cells, and has been implicated in cell-cycle progression [22]. Several nucleostemin-binding proteins have been identified, including p53, MDM2 and the telomeric repeat binding factor 1 (TRF1) [22], [25], [26]. Alterations in nucleostemin expression levels cause a decrease in the proliferation rate of cells, in both p53 dependent and independent manners, [22], [26]–[28]. The protein is indispensable for early embryogenesis [29] but is also important in adult neural stem cells [30]. Some studies have shown that depletion of nucleostemin is associated with a limited tumorigenic capacity in both HeLa and PC-3 cells [31], [24]. Beside its implication in the regulation of cell proliferation, several additional roles have been assigned to nucleostemin such as telomere length regulation by promoting the degradation of TRF1 [25], processing of pre-rRNAs [32] and maintenance of nucleolar architecture [33].

Our intention was to explore the role of nucleostemin in human GBM-CSCs using lentivirally transduced short hairpin RNAs (shRNAs) to severely reduce its presence in the cells. The CSCs depleted of nucleostemin expression (shRNA18) did not result in the profound reduction of cell proliferation and increased apoptosis that we had expected. Instead, an off-target lentivirus (shRNA22) abolished proliferation, self-renewal and survival of CSCs. Also, the presence of this shRNA significantly delayed CSCs tumorigenic capacity when xenografted in nude rat brains.

## Results

### Expression of nucleostemin in two human-glioblastoma-derived cancer stem cell lines

**Figure 1:**
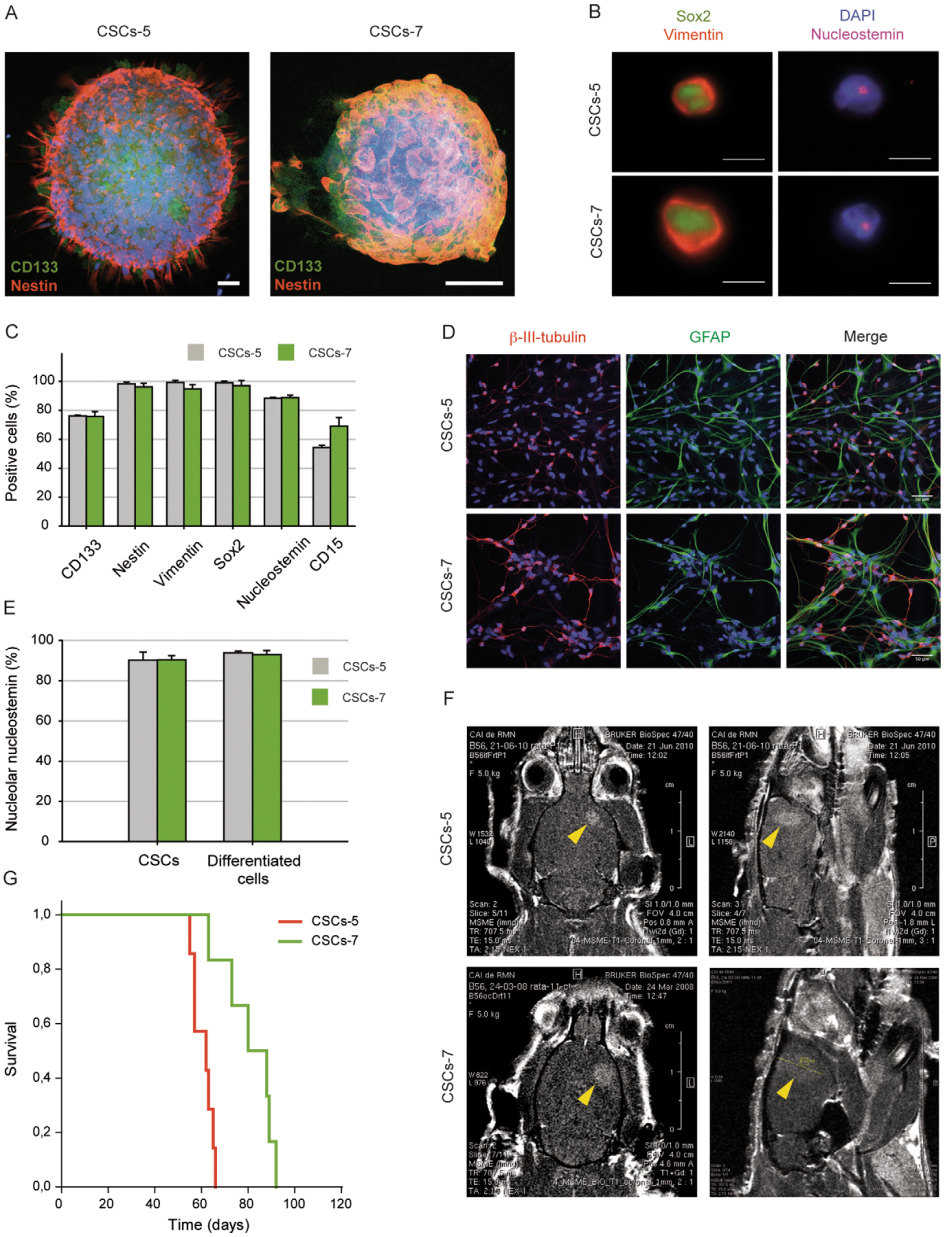
CSCs-5 and CSCs-7 characterization. **A.** CSCs-5 and CSCs-7 neurospheres expressing CD133 (green) and nestin (red). Scalebar: 50 µm. **B.** CSCs-5 and CSCs-7 cells showing the expression and cellular localization of Sox2 (green), vimentin (red) and nucleostemin (purple). Scalebar: 10 µm, and quantitative plot (**C**) of the stem cell markers CD133, nestin, vimentin, Sox2, nucleostemin and CD15 in CSCs-5 (gray) and CSCs-7 (green). **D.** Neuron (β-III-tubulin, pink-red) and astrocytic (GFAP, green) differentiation capacity of CSCs-5 and CSCs-7. Scalebar: 50 µm. **E.** Nucleolar nucleostemin expression in stem or differentiatiated CSCs-5 (gray) and CSCs-7 (green) cells. **F.** Magnetic resonance imaging of *in vivo* tumors developed from CSCs-5 and CSCs-7 (yellow arrowheads point to the tumor), and Kaplan-Meier survival analysis (**G**) of immunodeficent rats, after CSCs-5 (red) or CSC-7 (green) orthotopic xenografts.

Two cultures enriched for cancer stem cells were derived from human brain tumor specimens (samples CSCs-5 and CSCs-7). Both cell lines grew exponentially and formed neurospheres even when seeded at low density, indicating a strong self-renewal capacity. The neurospheres were positive to CD133 (Fig. 1A; green), nestin (Fig. 1A; red), Sox2 (Fig. 1B; green) vimentin (Fig. 1B; red), and nucleostemin (Fig. 1B; pink) neural stem cell markers. Nucleostemin was present in the nucleus of 88% of CSCs-5 and CSCs-7 cells, as determined by immunofluorescence assays (Fig. 1C). A large percentage of cells (76%) were also positive for CD133, and even higher percentages were obtained for nestin, vimentin and Sox2 (over 95%), the latter involved in self-renewal and proliferation of stem cells. CD15 was a marker for 54% of CSCs-5 and 69% for CSCs-7. The multipotency of these two cultures was demonstrated as the neurospheres grown in differentiation-inducing medium displayed typical morphological differentiation towards all the three neural lineages – astrocytic, neuronal and oligodendrocytic, as assessed by positivity for β-III-tubulin [34] and MAP2 (neuronal) (Fig. 1D red, and S1 red), GFAP (astrocytic) (Fig. 1D green), and NG2 (oligodendrocytic precursor) (Fig. S1 green) antigens respectively. Nevertheless, the differentiated cells did not loose expression of nucleostemin (Fig. 1E), despite not expressing any of the other stemness related genes (data not shown). The karyotypic analyses showed several alterations reflecting transforming activity, and the clonogenicity assays in soft agar indicated development of many colonies.

We then explored the potential of both CSCs-5 and CSCs-7 to form tumors after orthotopic xenogratf in immunodeficent rat brains. After injecting 5×10^5^ cells intra-cranially, both cell lines formed large tumors in 100% of the cases (n = 12 for CSCs-5 and 11 for CSCs-7), followed by magnetic resonance imaging (MRI) (Fig. 1F). The tumors were highly infiltrative (Fig. S2). Explants from these gliomas were serially transplanted into the brains of other nude rats and generated lethal tumors that were equally enriched in CSCs, demonstrating a high capacity for self-renewal. The mean survival times of the hosts were 60±4 days with CSCs-5 and 81±11 days for CSCs-7 cells (Fig. 1G). Histopathological analyses of xenografts showed the general characteristic features of GBM, as pseudopalisade and focal necrosis, high cellularity, high expression of EGFR and high proliferative index (MIB-1). Patients and xenografts samples show similar expression levels for all the markers, including medium (patient/xenograft 5) and low (patient/xenograft 7) expression for p53, and very low, if any, for p16 (Fig. S3). The results show that the transplanted tumors in the rats were phenocopies of the original patient tumors.

### Characterization of the shRNAs designed to target human nucleostemin

**Figure 2:**
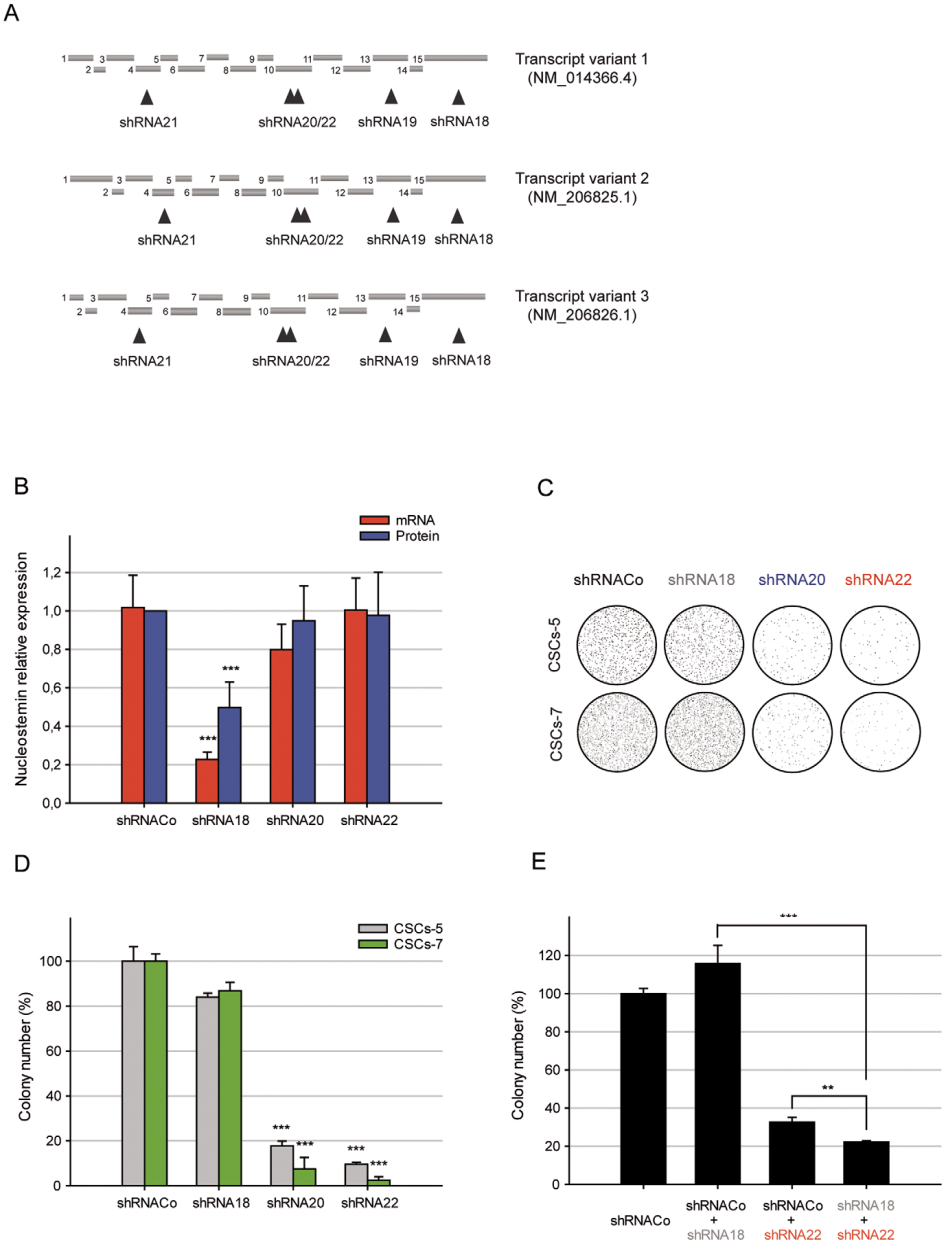
Nucleostemin-directed shRNAs effect in CSCs. **A.** Schematic representation of the 3 nucleostemin transcript variants and exons. Arrowheads point to each designed shRNA. **B.** Nucleostemin mRNA (red) and protein (blue) quantification in CSCs-5 treated with shRNACo, shRNA18, shRNA20 and shRNA22. **C.** shRNAs effect on CSCs-5 and CSCs-7 soft agar colony-forming ability, and its quantitative analysis (**D**). **E.** Soft agar colony counts in double-infections to determine the nucleostemin-specificity of shRNA22. ** p≤0.05; *** p≤0.001.

The nucleostemin gene has 15 exons, and as it undergoes alternative splicing produces three different mRNA transcripts. Except for the exon 1, these variants have similar sequences. Therefore, for designing specific primers to knock-down all three variants, we have chosen primers in common sites of exons 4, 10, 13 and 15 (Fig. 2A). We targeted nucleostemin by infection with lentivirus expressing an shRNA against the nucleolar protein. Five different shRNAs with perfect complementary sequences in the human nucleostemin mRNA were introduced to CSCs-5 and CSCs-7 cells. Three of the five shRNAs were chosen for further characterization: shRNA18, shRNA20 and shRNA22. The RT-PCR and western blot analysis revealed that while shRNA18 caused a significant reduction in nucleostemin mRNA (78%) and protein levels (51%), both shRNA20 and shRNA22 did not seem to affect the levels of the mRNA and protein, when normalized to the corresponding respective GAPDH (mRNA) and actin (protein) controls (Fig. 2B). The results suggest off-target binding of both shRNA20 and shRNA22. Alternatively, relatively minor reductions of nucleostemin messenger levels via target effect could have dominant effects on an assay if the output is very dose-sensitive to levels of this particular protein. We designed an assay, based upon colony formation in soft agar, to distinguish between the two possibilities. The expression of shRNA18 in CSCs-5 or CSCs-7 does not inhibit colony formation in soft agar, while expression of shRNA22, and to a lesser extent shRNA20, drastically reduced the number of colonies grown in this type of medium (Fig. 2C and 2D). If shRNA22 was causing a minor reduction in the nucleostemin message, and this triggered a major inhibition of growth because the pathway involved was very sensitive to minor level reductions of the messenger or protein, we should expect a similar effect when cells are either infected with shRNA18 alone, or when cells are simultaneous infected with both shRNA18 and shRNA22. That is, a large number of colonies growing. Conversely, if a true off-target effect were operating here, the result of this combination would be the opposite, as the real target would be different from nucleostemin, and we would observe as few colonies growing in soft agar as the ones observed when shRNA22 was used alone. The infection of cells with a lentivirus not carrying an shRNA was used as a control. The measurement of the number of CSCs-5 colonies formed in soft agar after different combined treatments gave the following results (Fig. 2E): a high number of colonies for the control shRNA (shRNACo) and for the combination shRNACo+shRNA18, as expected, and a low number of colonies for both shRNACo+shRNA22, and shRNA18+shRNA22. Performing this experiment, we were able to discriminate between the possibilities of all shRNAs targeting nucleostemin to different degrees or, the shRNA22 having a different target. If the effect was due to the low level of inhibition, the co-expression of the shRNA18 and shRNA22 should mask the effect of the latter, since shRNA18 would inhibit stronger the expression of the nucleostemin gene. On the other hand, if the effect was off-target, we would appreciate the consequences of shRNA22 independently of the expression of shRNA18. The results show clearly that shRNA22 induces his effect independently of the expression of shRNA18, indicating that is due to an off-target effect. We therefore ruled out the hypothesis of a very minor reduction of nucleostemin by shRNA22 having a dominant negative effect in cell growth. We believe that the growth inhibition effect of shRNA22 in soft agar is due to a *bona fide* off target effect.

**Figure 3:**
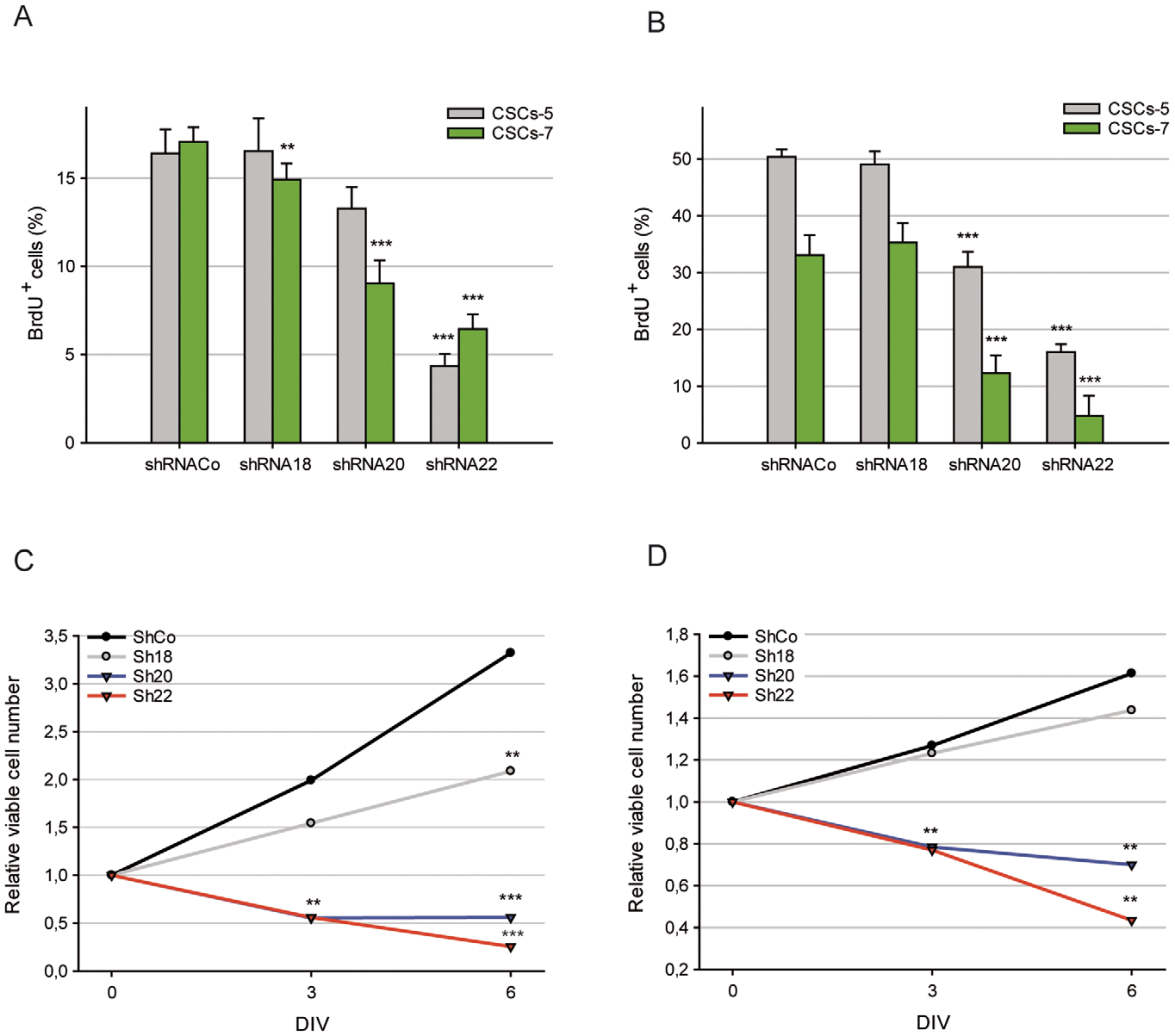
shRNAs effect in the proliferation kinetic of CSCs. **A.** Number of phase-S or total-cycling (**B**) CSCs-5 (grey) and CSCs-7 (green) cells treated with the different shRNAs. **C.** Number of viable cells on DIV 0, 3 and 6 in CSCs-5 and CSCs-7 (**D**) treated with the different shRNAs. ** p≤0.05; *** p≤0.001.

Depletion of nucleostemin by shRNA18, did not significantly reduce the S phase cell population as indicated by the level of BrdU uptake after a 15 min-pulse (Fig. 3A) or the total population of cells cycling captured by a 20 h BrdU treatment (Fig. 3B). The other two shRNAs: shRNA20 and shRNA22, having a very low, if any, inhibition of expression, displayed a consistently lower percentage of S phase and cycling cells. As demonstrated by growth curve assays, the total number of cells infected with the control lentivirus lacking shRNA-expressing insert (shRNACo) increased several fold over six days. The shRNA20 or shRNA22 infected cells did not increase in number, or even decrease considerably (Fig. 3C and 3D), while CSCs infected with shRNA18 behaved much closer to control cells than to shRNA20 and shRNA22. The results suggest an implication of the primary target of shRNA22, and to a slightly lesser extent to the shRNA20 hit, in sustaining growth and survival of human GBM-CSCs. We are not certain about shRNA20 and shRNA22 sharing a primary target, despite the physical closeness of the original shRNA20 and shRNA22 sequences in the nucleostemin gene. Although only 66 nucleotides separate these two sequences, a human sequence alignment search taking into account the 66 nucleotides between them did not reveal any match besides nucleostemin.

### The off-target shRNA20 and shRNA22, induce apoptosis preferentially in CD133+ CSCs and impairs neurosphere formation

**Figure 4:**
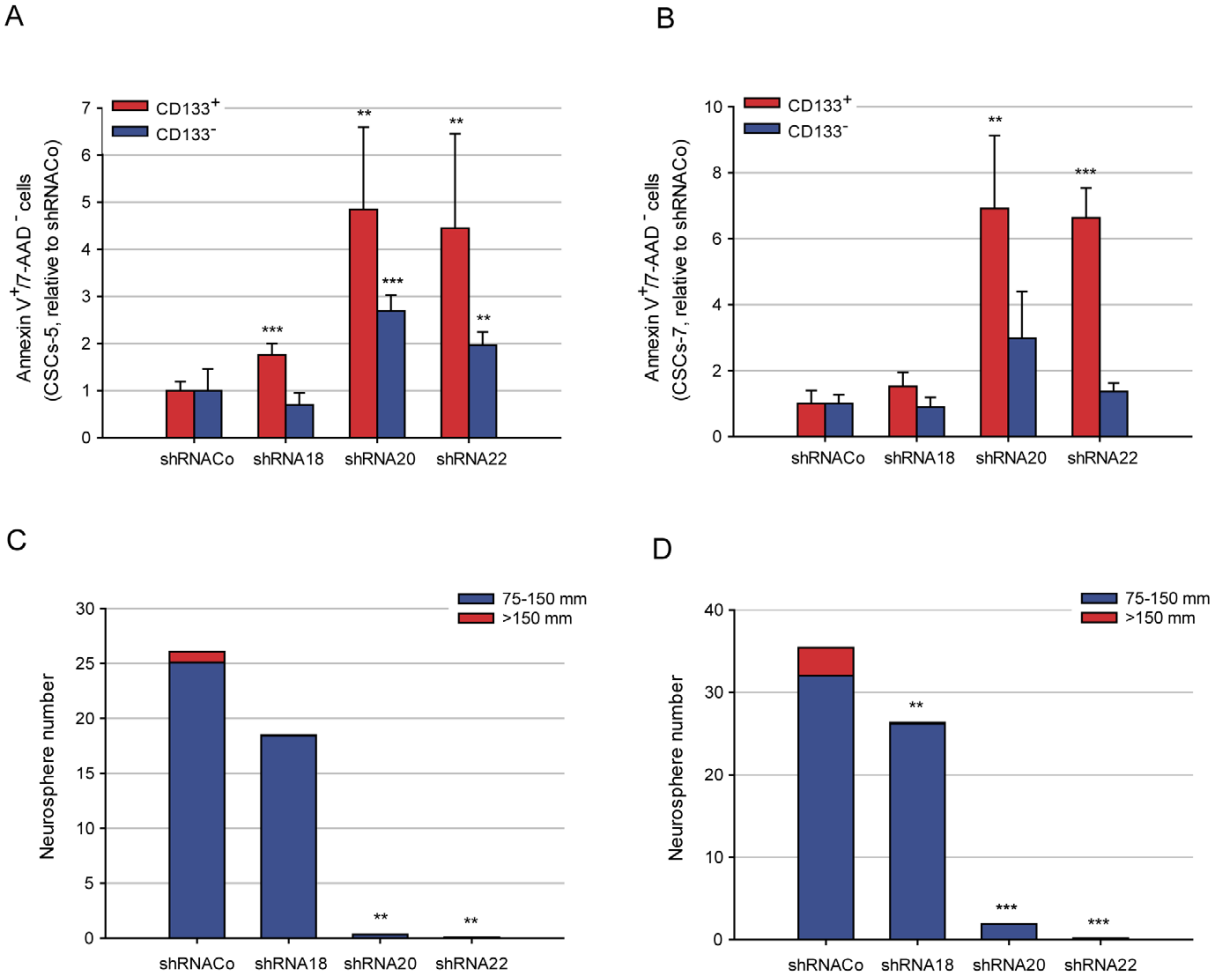
shRNAs effect in the viability of CSCs. **A.** Apoptosis induced by shRNAs in CSCs-5 and CSCs-7 (**B**) CD133^+^ (red) or CD133^−^ (blue) populations. **C.** Neurosphere forming-ability of CSCs-5 and CSCs-7 (**D**) cells, treated with the different shRNAs. ** p≤0.05; *** p≤0.001.

The presence of these shRNAs affect not only cell proliferation but also cellular survival. We quantified the percentage of apoptotic cells in both CSCs-5 and 7 populations following shRNACo, shRNA18, shRNA20 and shRNA22 lentiviral infection. 7-AAD/annexin V-staining revealed a preferential apoptosis in CD133^+^ cells in both CSC-5 and 7 populations (Fig. 4A and 4B); the relative values with respect to the controls for CSCs-5 and CSCs-7 are shown in Table 1. Also the percentage of CD133^+^ cells is reduced in both CSCs-5 and CSCs-7 populations following shRNA20 and shRNA22 lentiviral infection (Fig. S4). These results suggest that the primary target of shRNA22 and shRNA20 is a survival factor for GBM-CSCs preferentially expressed in CD133^+^ cells.

**Table 1:** Preferential apoptosis in CD133^+^ cells in both CSC-5 and CSC-7 cultures.

	CD133^+^		CD133^−^	
CSCs-5	Mean	s.d.	Mean	s.d.
ShRNACo	1	0,1915	1	0,4633
ShRNA18	1,7582	0,2432	0,7005	0,2498
ShRNA20	4,8462	1,7494	2,6928	0,3333
ShRNA22	4,4463	2,009	1,968	0,2748
**CSCs-7**				
ShRNACo	1	0,3987	1	0,264
ShRNA18	1,5165	0,4393	0,8947	0,2959
ShRNA20	6,9138	2,2152	2,9816	1,4131
ShRNA22	6,6357	0,8982	1,3607	0,2593

Sharing certain key characteristics of normal stem cells, CSCs are capable of self-renewal, which allows sustained maintenance of this subpopulation and expansion of the corresponding tumor. Neurosphere formation capacity after treatment with the different shRNAs was measured (Fig. 4C and 4D). Practically no spheres were formed by cells treated with shRNA22 and very few by ShRNA20 treated cells in both CSCs-5 and 7 populations. Neurospheres developed in 100% of the control groups. These results, together with our observations that CSCs failed to proliferate and underwent apoptosis, suggest that the primary target of shRNA22 and shRNA20 is required for self-renewal of glioblastoma cancer stem cells.

### The off-target shRNA22 significantly reduces the tumorigenic potential of CSCs-5

**Figure 5:**
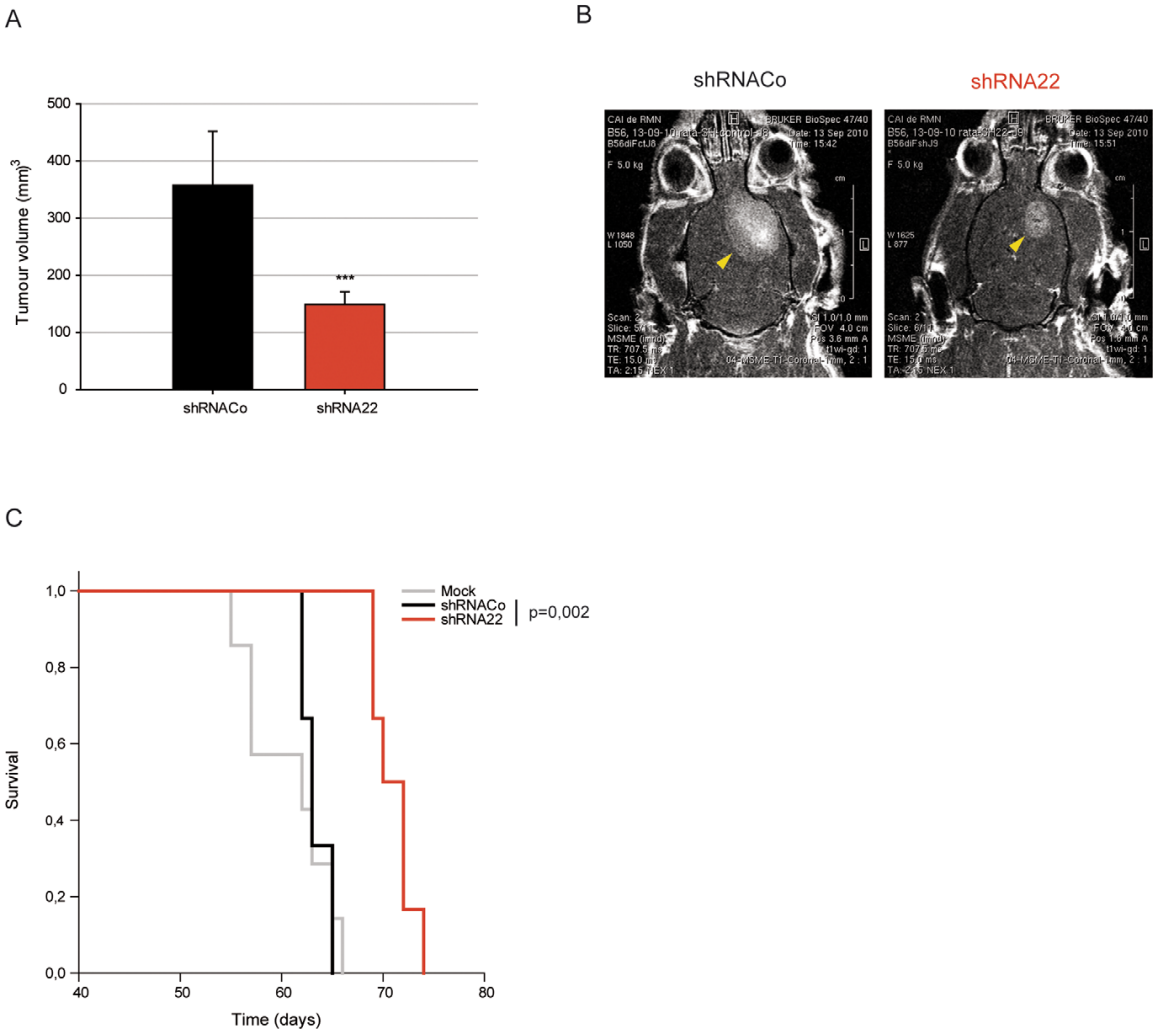
shRNAs effect in the in vivo tumor-development of the CSCs-5. **A.** Quantification of the volumes of tumors induced with CSCs-5 cells treated with shRNACo (black) and shRNA22 (red). **B.** Magnetic resonance imaging of representative examples of tumors after orthotopic xenografts in nude rat-brains of CSCs-5 cells carrying shRNACo or shRNA22, and Kaplan-Meier survival plot (**C**) of the immunodeficent rats inoculated with CSCs-5 (mock, gray), cells carrying shRNACo (black) and shRNA22 (red). *** p≤0.001.

We examined the effect of infecting CSCs-5 cells with control lentiviruses or lentiviruses expressing shRNA22. Following puromycin selection, 5×10^5^ cells were injected into the brains of nude rats. All animals bearing control cells, that is cells infected with empty lentivirus, (n = 3) developed large tumors, followed by magnetic resonance, and died in 63±1 days, not very different from uninfected CSCs-5 (61±4 days, n = 12). In contrast, the animals injected with cells expressing shRNA22 (n = 6), developed significantly smaller tumors, being the median volume of 148 mm^3^, while the mean value of tumors expressing shRNACo were 358 mm^3^ (Fig. 5A and 5B). The Kaplan-Meier survival plot indicates a significant difference between the shRNACo and shRNA22 treated tumoral cells, with a mean survival of the latter of 71±2 days (Fig. 5C). Thus, the presence and expression of shRNA22 in CSCs appears to be important for attenuating tumor progression.

### The effect of shRNA22 does not seem to be exclusive for CSCs

**Figure 6:**
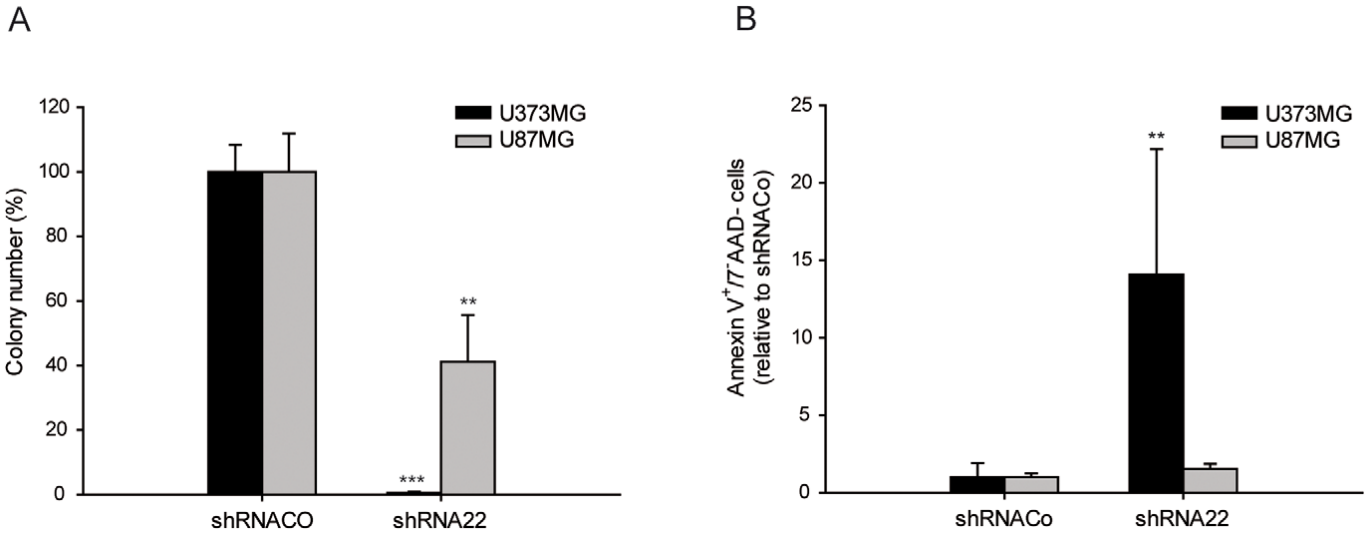
shRNAs effect in U373MG and U87MG cell lines. **A.** shRNAs effect on U373MG (black) and U87MG (gray) soft agar colony-forming ability. **B.** shRNAs-induced apoptosis in U373MG (black) and U87MG (gray). ** p≤0.05; *** p≤0.001.

We attempted to determine the effect of shRNA22 in glioma cells with no CD133^+^ stem properties such as U87MG and U373MG human glioma cell lines. We assessed their clonogenic potential by soft agar assay following shRNACo and shRNA22 lentiviral infection. A significant reduction in the average number of colonies was observed when shRNA22 was expressed in U373MG (down to 1%), and to a lesser extent when in the U87MG glioma cell line (down to 41%) (Fig. 6A). We also measured cellular survival by estimating the percentage of apoptotic cells in both U373MG and U87MG cell lines following shRNACo, and shRNA22 lentiviral infection. 7-AAD/annexin V-staining revealed preferential apoptosis in U373 cells in a similar behaviour to CSCs. On the other hand, U87MG cells appeared rather resistant to apoptosis (Fig. 6B). The results suggest that although shRNA22 may attenuate tumoral growth in glioma cells in general, its impact may depend upon the cell type. The finding that shRNA22 effect is not limited to CSCs is important when designing strategies to eliminate all cancer cell types that build up a highly heterogeneous tumor such as glioblastoma.

### Attempts to identify the primary target of shRNA22

We carried out a genomic search for sequences, other than nucleostemin, with different degrees of homology with shRNA22 nucleotides. For this we used the basic local alignment search tool (BLAST) and we select four candidates: PCLO, involved in neurotransmisor release; HSPA13, member of the chaperon family HSP70; SEC22C, involved in vesicular traffic; and CNOT1 related to transcription and mRNA degradation. Of the 21 nucleotides, 14 were a perfect match in all cases except CNOT, with a 13 nucleotides match. However, not one of the four candidates was the primary target for shRNA22 as measured by qRT-PCR (data not shown) as no differences were observed in the concentration of their mRNA's in the presence or absence of shRNA22 related to shRNACo.

**Figure 7:**
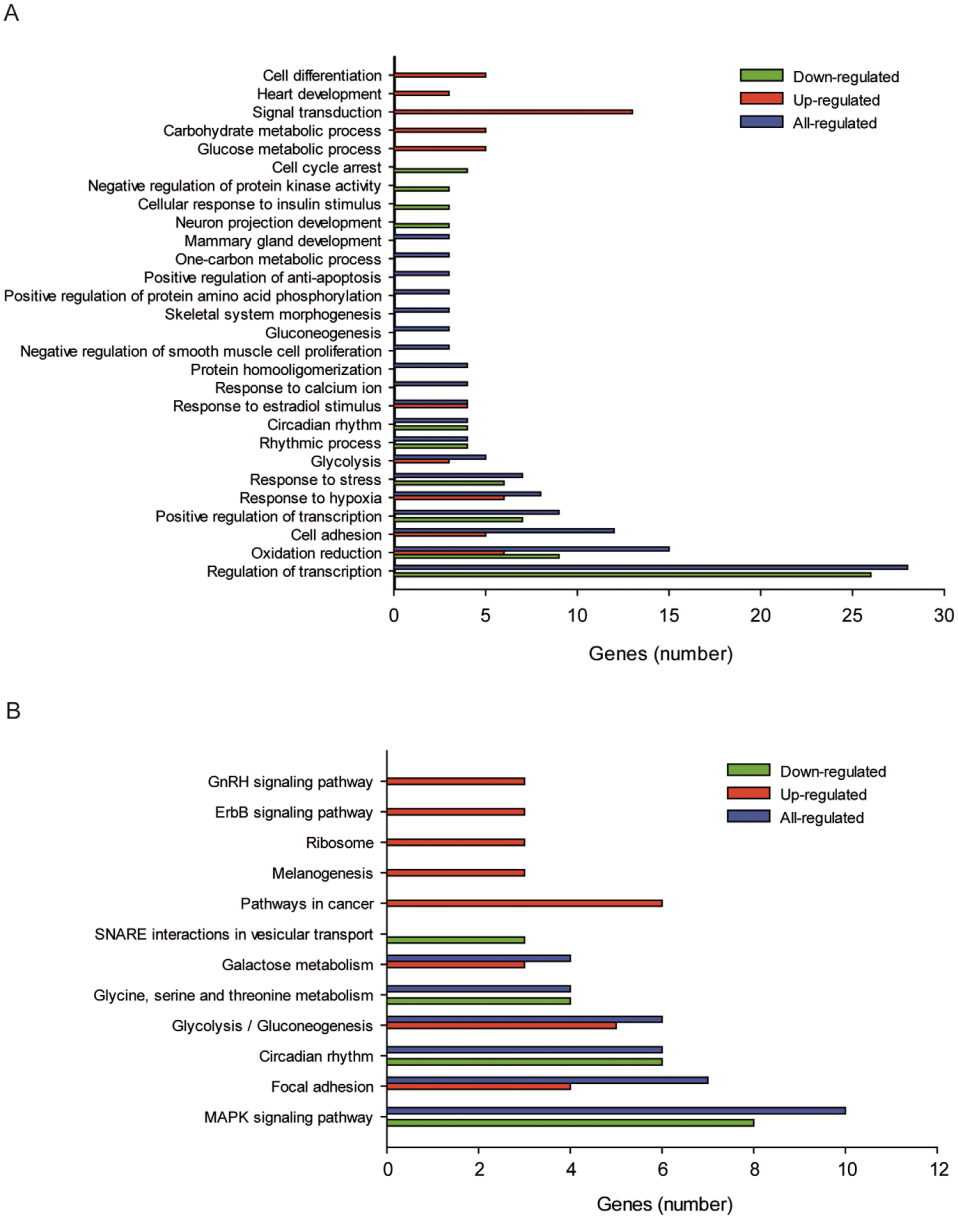
Gene expression profiling in the presence and absence of shRNA22. **A.** Significative down- (green), up- (red) and all-regulated (blue) genes grouped by function of biological processes, or by belonging to signaling pathways (**B**) induced by shRNA22 in CSCs-5 cells.

To determine genome-wide differentially expressed genes in CSCs-5 after shRNA22 infection, we used Affymetrix GeneChip Gene 1.0 ST Array System containing approximately 28.869 human genes, including the 3′ untranslated region (UTR) of the mRNAs that could be a target for binding in a micro (mi)RNA-like manner. As a result we identified 182 genes down-regulated in CSCs-5 treated with shRNA22 in relation to shRNACo (Table S1). As expected, nucleostemin was not among the down-regulated genes. Attempts to group the genes with shRNA22 hits by function (based on Gene Ontology and signaling pathway data) did reveal the presence of 26 genes involved in regulation of transcription among the silenced genes, and 56 DNA binding proteins (Fig. 7A). The signal transduction pathway with more genes down-regulated was the MAPK kinases pathway (Fig. 7B). Although the results so far are not conclusive, an indication that shRNA22 may be involved in silencing a transcription factor implicated in one of the MAP-kinases signaling-pathways can be suggested. Alternatively, multiple target effects are also a strong possibility for shRNA22, in a similar fashion to micro RNAs.

## Discussion

The similarities and differences between SCs and CSCs have been the source of much contention [35], [36], [37]. Brain CSCs resemble neural SCs in terms of phenotype, signaling and behaviour in vitro [38], but it is currently unclear whether the CSCs are, in fact, *bona fide* stem cells. Experimental characterizations of cancer stem cell populations may help in these matters. In rats, nucleostemin is highly expressed in the nucleoli of central nervous system SCs, but not in their differentiated progeny, as the gene seems to be abruptly shut off during differentiation prior to terminal stages [22]. Our observations of nucleostemin being expressed even after CSCs were forced to differentiate in culture, implies a new signature for CSCs and a significant difference with SCs, not previously reported.

We used an RNAi approach to specifically promote degradation of nucleostemin mRNA. Previous data has shown that knocking-down nucleostemin expression caused a severe decline in cell proliferation in bladder cancer cells [39] and reduced the sphere-forming ability in human breast cancer stem cells [40]. A significant reduction in cell-cycle progression in different types of human brain tumors and in human glioblastoma derived cell lines was also reported [41]. However, we found that the only shRNAi that efficiently reduces the protein, does not suppress cell cycle progression, does not decrease growth of CSCs cultures, and does not decrease the number of colonies formed in soft agar, as compared to controls infected with an empty lentivirus. The discrepancies could be due to differences in the knocking-down levels of nucleostemin achieved in previous reports (higher than 75% and sometimes more than 90%) and us (51%).

RNAi technology has been widely used in mammalian cells to suppress the expression level of individual genes, thus helping to define the functional roles of genes, particularly in diseases. Much work has centered around shRNA design algorithms, with a focus on gene-target specificity and efficiency [42], [43]. Nevertheless off-target effects are widespread, and individual shRNAs have been shown to down-regulate one or several other “unwanted” genes [44], [45], sometimes by binding in a micro (mi)RNA-like manner to the 3′ UTRs of mRNAs [46]. We found than shRNA22 is binding to other/s than the intended target gene. Off-target effects reported in previous RNAi studies were mediated by partial complementarity between shRNAs and the 3′ UTRs of off-target genes, involving a heptamer or hexamer ‘seed’ match of the shRNA strand at the 5′ end at positions 2 to 8 or 2 to 7, respectively [46], [47]. The mechanism is similar to that of miRNAs. Although the BLAST library used included the 3′UTRs of the messengers, a 7-nucleotide match parameter was not included in the screen analysis, as it would represent a severe relaxation of the conditions and a large increase in putative matches. A common primary target for shRNA20 and shRNA22 is presumed, as the sequences are only 66 nucleotides apart in nucleostemin exon 10. It is possible that a nucleostemin sequence including the shRNA22, the region between the two shRNAs and shRNA20 (maybe partially), would also be present in another genomic region, coding for a different protein whose mRNA secondary structure would be more accessible to shRNA22 and shRNA20 than the one at the nucleostemin mRNA. Nevertheless, no new matches beside nucleostemin were found in a human genome-wide search for complementary regions to the two-shRNA sequences and the nucleotides between them, performed using the basic local alignment search tool (BLAST).

The gene expression profiling of CSCs-5 in the presence or absence of shRNA22 did not unequivocally indicate its primary target. Rather, multiple target effects are a reasonable possibility for this shRNA. The largest number of genes silenced was related to the regulation of transcription when hits were grouped by function, and over-representation of genes involved in the MAPK signaling pathway were down-regulated by shRNA22 expression. Over-expression of one or more genes of the MAPK signaling pathway is common in glioblastomas, and several small molecules that inhibit the PI3 kinase-Akt signaling pathway are in clinical development. Although many of these molecules have been effective in preclinical models, it remains unclear whether this strategy alone will be sufficient to interrupt the molecular events initiated and maintained by signaling along the pathways because of the activation of other pathways that compensate for the inhibition of the targeted kinase. Attempts have recently been made to identify genes or pathways whose inactivation, in combination with the PI3K inhibitors PX-866 and NVPBEZ-235, might result in a lethal phenotype in glioblastoma multiforme cells [48]. The identified shRNA22, when expressed in CSCs from glioblastoma patients, inhibits cell proliferation and self-renewal, induces apoptosis and significantly reduces their tumorigenic potential when xenotransplanted into the brains of nude rats. The fact that the primary target of shRNA22 is not limited to CSCs is important, because when trying to eliminate a tumor one must also target the tumor stroma cells and microglia because these cells contribute to tumor maintenance and progression. The use of this shRNA alone or in combination with other drugs may represent a potential clinical therapeutic strategy.

## Materials and Methods

### Statement for animal care

This study was carried out in strict accordance with the legislation and guidelines for animal care and handling in Spain (Spanish Royal Decree 1201/2005 BOE published October 21st 2005), and those from the European Union (2003/65/CE from the European Parliament and Council July 2003). The protocol was approved by the Committee on the Ethics of Animal Experiments of the Autonomous University of Madrid (permit associated to project SAF 2009-07259 issued in Madrid 11^th^ March 2009) and by the CBMSO institutional Biosafety Committee. All surgery was performed under isofluorane gas anesthesia, and all efforts were made to minimize suffering.

### Statement for patient Consent

We obtained two new cancer stem cells lines (CSCs-5 and CSCs-7) from primary glioblastoma surgical specimens from patients undergoing resection for newly diagnosed glioma in accordance with protocols approved by the Institutional Ethic Committee of the Ramón y Cajal Hospital. Written informed consent to utilize excess tissue for research was obtained from each patient, and de-identified tissues, to protect anonymity, were used (permit associated to project SAF 2009-07259, issued in Madrid the 26^th^ February 2009, Spain).

### Statement for microarray data

All data presented here is MIAME compliant and the raw data has been deposited in the GEO compliant database (Access number: GSE30448).

### Cell isolation and culture

The new cultures, CSCs-5 and CSCs-7, were established from fresh surgical specimens collected directly from the operating theatre in PBS plus 0,6% glucose and immediately transported on ice to the cell culture room and processed as follows: the tumour pieces were washed and minced finely with small scissors, they were then incubated in 0,1% trypsin and 0,04% DNase (type II, Sigma) in PBS for 1 h at 37°C. Digested tissue was washed two times and mechanically dissociated by passing through fire-polished Pasteur pippetes. Finally, the cell suspension obtained was filtered in a 40 µm cell strainer (BD). The number and viability of the cells were measured with trypan blue assay. Neurosphere culture was initiated by seeding cells at a density of 1×10^5^ viable cells/ml in a proliferation medium consisting of DMEM∶F12 (1∶1) supplemented with glutamax (Gibco), 0,5% albumax I (Gibco), 5 mM hepes (Gibco), 0,6% glucose (Sigma), N2 (1×, Invitrogen), 2 µg/ml heparin (Sigma), 20 ng/ml of both EGF and FGF-2 (Peprotech), aminoacids (L-Ala 44 mM, L-Asn 45 mM, L-Asp 40 mM, L-Glu 40 mM, L-Pro 30 mM) and gentamicine (0,055 mg/ml). The cultures were grown at 37°C in a 97% humidity atmosphere containing 5% CO_2_, and fed twice a week with a 20% volume of fresh medium. In these conditions, neurospheres developed in one to two weeks. When the size reached 200–300 µm, the neurospheres were dissociated with trypsin-EDTA and re-seeded. U87MG [46] and U373MG [46] cell lines were grown in DMEM (Gibco) supplemented with 10% foetal bovine serum (Sigma).

### Lentiviral vector production and infection

The shRNA lentiviral vectors against nucleostemin were commercially obtained from Sigma-Aldrich; (MISSION shRNA TRCN0000074218, TRCN0000074219, TRCN0000074220, TRCN0000074221 and TRCN0000074222). We used pLKO.1 as negative control, with no shRNA insert (SHC001, Sigma-Aldrich). The vectors were co-transfected with pCMV-dR8.74 packaging plasmid and pMD2G envelope plasmid into 293T [46] cells with optimem, lipofectamine and plus reagent (Invitrogen), to produce virus. After 24 h, the optimem was changed to CSC medium, the cells were incubated for 24 h and then the supernatants were collected, filtered and stored at −80°C. For infections, 6×10^4^ viable cells/cm^2^ were seeded into optimem medium and incubated for 6 h. Then, the viral particles (1 ml of collected supernatant per P100 plate) and 4 µg/ml polybrene were added and incubated over night. The optimem was changed to proliferation medium and the cells were incubated for 8 h prior puromycin treatment (0,8 µg/ml). The cells were incubated for an additional 96 h to insure that only the infected cells were alive.

### Immunocitochemistry

The cells were attached onto poli-lysine (0,0033%, Sigma) and laminine (10 µg/ml, sigma) coated glass coverslips and fixed with 4% parafolmaldehyde. They were then permeabilizated for 10 min with 0,1% Triton X-100 in PBS and blocked for 20 min with 0,1% Triton X-100 and 1% FBS in PBS. The followings antibodies were incubated over night at 4°C: rabbit anti-CD133 (1∶100, Abcam), rabbit anti-hNS (1∶500, Chemicon), mouse anti-vimentin (1∶200, Roche), rabbit anti-Sox2 (1∶200, Chemicon) and rabbit anti-NG2 (1∶200, Chemicon). The following antibodies were incubated for 1 h at room temeperature: mouse anti-nestin (1∶500, Chemicon), mouse anti-β-III-tubulin (1∶2000, Promega), mouse anti-MAP2 (1∶1000, Sigma), and rabbit anti-GFAP (1∶1000, Promega). The coverslips were washed three times and incubated for 1 h at room temperature with the appropriate secondary antibody: donkey anti-rabbit IgGs Alexa 488 (1∶500, Invitrogen) or donkey anti-mouse IgGs Alexa 555 (1∶500, Invitrogen). For BrdU labeling, a 20 h pulse of 10 µM BrdU (to mark all cycling cells) or 15 min of 40 µM BrdU (to mark S-phase cells) was used. The cells were fixed, made permeable with 0,2% Triton X-100 in PBS for 20 min, treated with HCl 1 M for 10 min and HCl 2 M for 20 min, neutralized with Na_2_B_4_O_7_ 0,1 M pH 8,5 for 2 min and blocked with 0,2% Triton X-100 and 1% FBS in PBS for 20 min. Mouse anti-BrdU antibody (1∶100, BD) were incubated for 1 h at room temperature. In all cases, the nuclei were counterstained with DAPI. For fluorescence imaging, we used Axiovert200 microscope (Zeiss).

### Immunohistochemistry

The tissues were fixed with PFA 4%, paraffin-embedded, and chopped in to 5 µm slides. The samples were de-paraffinized and hydrated by sequential washes of 5 min (twice each) in xylene, absolute ethanol, 95% ethanol, 70% ethanol and water. Then, antigens were retrieved incubating the samples in citrate 10 mM pH 6 during 10 min at 95°C, cooling down to RT and washing twice with water and PBS. They were then blocked for 1 hour with 0,3% Triton X-100 and 5% FBS in PBS. The mouse anti-human nestin (1∶500, Chemicon) antibody was incubated for 1 h at room temperature. The samples were washed three times and incubated for 1 h at room temperature with the donkey anti-mouse IgGs Alexa 488 (1∶500, Invitrogen) secondary antibody. The nuclei were counterstained with DAPI.

### Soft agar assay

To evaluate the tumorigenic potential of the cells after the inhibition of nucleostemin, 2×10^4^ viable cells per well were plated in soft agar in 6-wells plates. Briefly, the base layer was made by mixing equal volumes of sterile 1% agar, cooled to 40°C, and 2× proliferation medium, to obtain a final solution of 0,5% agar in 1× CSC medium. For the top layer, the agar was diluted to 0,7% in distilled water, cooled to 40°C and then mixed in equal proportions with 2× CSC medium. The cells were immediately added to the mix to yield a final solution of 0,35% agar in 1× CSC medium containing 10.000 cells/ml. No selection agent was added for this assay. The cells grew for 14 days at 37°C in a humidified atmosphere containing 5% CO2 and then viable colonies were stained with 1 ml/well of 600 µg/ml MTT (Thiazolyl Blue Tetrazolium Bromide, Sigma), photographed, and counted using ImageJ software (http://rsbweb.nih.gov/ij/).

### Secondary neurosphere formation assay

Infected cells were plated at low density (1×10^4^ viable cells/ml) in 96-well plates in proliferation medium with 0,8 µg/ml puromycin and grew for 9 generations. Then, the wells were photographed in a bright field with an Axiovert200 microscope (Zeiss) and visually scored for the number and size of the spheres generated in each case. ImageJ software was used to set the scale of the image.

### Flow cytometry

A single cell suspension was resuspended in PBS and labeled with 5 µl per million cells using CD133/2(292C3)-APC antibody (Miltenyi Biotec) or 10 µl per million cells for the isotype control antibody (mouse IgG2b-APC, Immunostep research), according to the manufacturer's instructions, and analyzed using a FACSCalibur (Beckton Dickinson). If necessary, a sequential stain was made to assay the apoptosis. CD133 marked cells were washed with 0,5 ml of binding buffer (10 mM Hepes pH 7,4; 140 mM NaCl, 2,5 mM CaCl) and resuspended in 200 µl of binding buffer plus 6 µl Annexin V-FITC (BD Pharmingen) and 5 µl 7-amino-actinomycin D (7-AAD, BD Pharmingen), incubated for 15 min at room temperature and immediately analyzed. The data collected was analyzed with FlowJo software.

### Real-time quantitative PCR

Cell pellets were collected by centrifugation at 7500 rpm for 5 min at 4°C. The RNA was extracted using TRIzol reagent (Invitrogen) and analyzed by electrophoresis. The concentration was estimated using a Nanodrop ND-100 (Thermo Scientific). cDNA from total RNA (1 µg per sample) was synthesized using the *High capacity cDNA achieve* (Applied Biosystems). Equal amounts of cDNA from each sample were amplified with nucleostemin TaqMan probe (Hs01015887_g1, Applied Biosystems). A TaqMan probe of human house keeping GAPDH (Hs 99999905_m1, Applied Biosystems) was used for internal control.

### Western blot

The cells were lisated in lysis buffer (50 mM Tris-HCl pH 7,5; 300 mM NaCl, 0,5% SDS, 1% Triton X-100 and 10 µM PMSF). Protein concentration was measured by *Dc protein assay* (BioRad). Proteins (25 µg) were separated in 12% SDS-polyacrylamide gel and transferred to nitrocellulose membranes (Whattman). The membranes were blocked with 5% fat-free milk powder and 0,3% tween-20 (Merck) for 1 h at room temperature. The rabbit anti-hNS (1∶5000, Chemicon) and rabbit anti-actin (1∶1000, Sigma) were incubated for 1 h at room temperature. Membranes were washed four times in 0,3% tween-20 in PBS and incubated for 1 h at room temperature with the secondary antibody (rabbit Ig-HPR, 1∶3000, DAKO). Immunoblots were developed and densitometric analyses were performed using Quantity One software (Bio-Rad).

### Intracraneal xenograft

5×10^5^ viable cells, infected and selected, were stereotactically implanted in 5 µl of PBS to generate orthotopic xenografts as follows: Nude rats (rnu/rnu, Charles River) were immobilized on a stereotaxic frame (Kopf) and anaesthetized with a constant flow of isoflurane, O_2_ (2 l/min) and N_2_O (1 l/min). A longitudinal incision was made and small craneotomy was performed at 4 mm to the right of *bregma*. The cells were injected at 4 mm depth from the dural membrane with a Hamilton syringe and an automatic injector (Stoelting Mod.310) at a rate of 1 µl/min. The surgical field was cleaned and closed with a surgical stapler.

### Microarray analysis

CSCs-5 cells were infected with shRNACo and shRNA22 as previously described, and exposed to puromycin for 62 h. Then, the RNA was extracted by RNeasy kit (Quiagen) and its quality was measured using a 2100B Bioanalyzer (Agilent Technologies). The cDNA was synthesized using the *High capacity cDNA achieve* (Applied Biosystems). We chosen the GeneChip Gene 1.0 ST Array System for human (Affymetrix), performed in the Genomic unit of the Parque Científico de Madrid, following the manufacturer's protocol. Gene ontology analysis was performed by the same unit using GeneCoDis (http://genecodis.dacya.ucm.es/).

### Statistical analysis

The statistical analysis was performed using a two-tailed Student's *t* test (unpaired). Survival analysis of in vivo experiments was performed by Kaplan-Meier curves.

## Supporting Information

Figure S1 **MAP2, GFAP and NG2 expression.** Differentiated CSCs-5 and CSCs-7 cells showing the neuronal MAP2 (red, upper panels), astrocytic GFAP (green, upper panels), and oligodendrocytic precursor NG2 (green, lower panels) markers. Scalebar: 50 µm.
(TIF)

Figure S2 **Invasiveness of tumors.** **A.** Magnetic resonance imaging showing the infiltrative capacity of the CSCs-5-induced tumors. Asterisks: inoculation points; arrowheads: tumoral tissue limit. **B.** Contralateral hemisphere invasion of CSCs-5 cells. **C.** As a contrast, a much better delimited and less invasive tumor induced by U87MG cells. **D.** GBM invasiveness confirmation by histology in both patient-xenograft pairs. Hematoxylin and eosin staining. 10× objective. **E.** Pathologic study showing the pseudopalisading formation and necrosis detail (whiter areas) in patient and xenograft-7. 10× objective. **F.** Specific anti-human nestin staining (green) in xenograft 5 showing GBM cells infiltrating the cerebral parenchyma. Scale bar indicates 500 µm in the left and center panels, and 250 µm in the right panel.
(TIF)

Figure S3 **Histopathological analysis of both patients and xenografts tumors.** Comparison between xenografts of both patient-derived CSCs and the original tumors of haematoxilin-eosin staining, p53, p16 and EGFR expression, and the proliferative index (MIB-1).
(TIF)

Figure S4 **Death of CD133^+^ cells.** **A.** Percentage of CD133^+^ cells in CSCs-5 and CSCs-7 (**B**) cells treated with the different shRNAs.
(TIF)

Table S1 Names of the down-regulated genes when cells are expressing shRNA22, ratio shRNA22/shRNACo, p-value t. test, gene symbol and mRNA accession-number.
(DOCX)
