# Supplementary figures and images for: An Off-Target Nucleostemin RNAi Inhibits Growth in Human Glioblastoma-Derived Cancer Stem Cells
Source: PLoS One. 2011 Dec 12;6(12):e28753. doi: 10.1371/journal.pone.0028753 (PMC3236221; doi:10.1371/journal.pone.0028753)

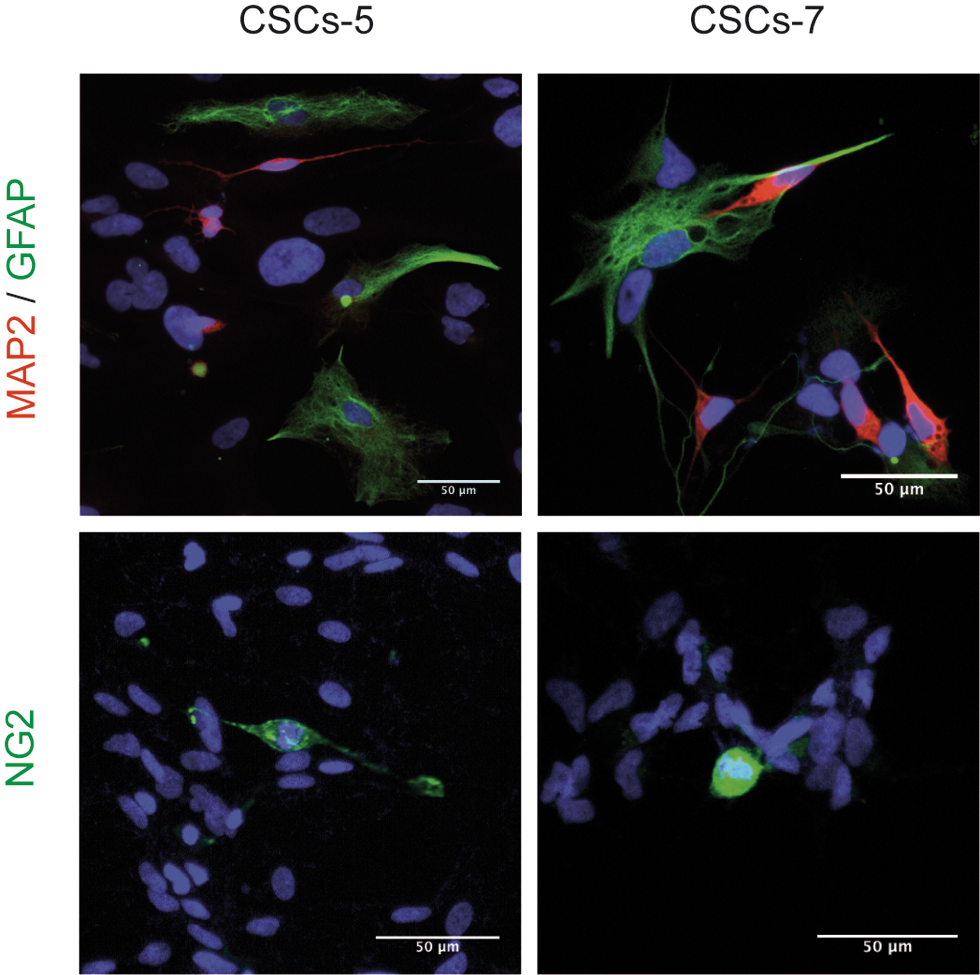

Supplement: Figure S1 — MAP2, GFAP and NG2 expression. Differentiated CSCs-5 and CSCs-7 cells showing the neuronal MAP2 (red, upper panels), astrocytic GFAP (green, upper panels), and oligodendrocytic precursor NG2 (green, lower panels) markers. Scalebar: 50 µm. (TIF) [file pone.0028753.s001.tif]

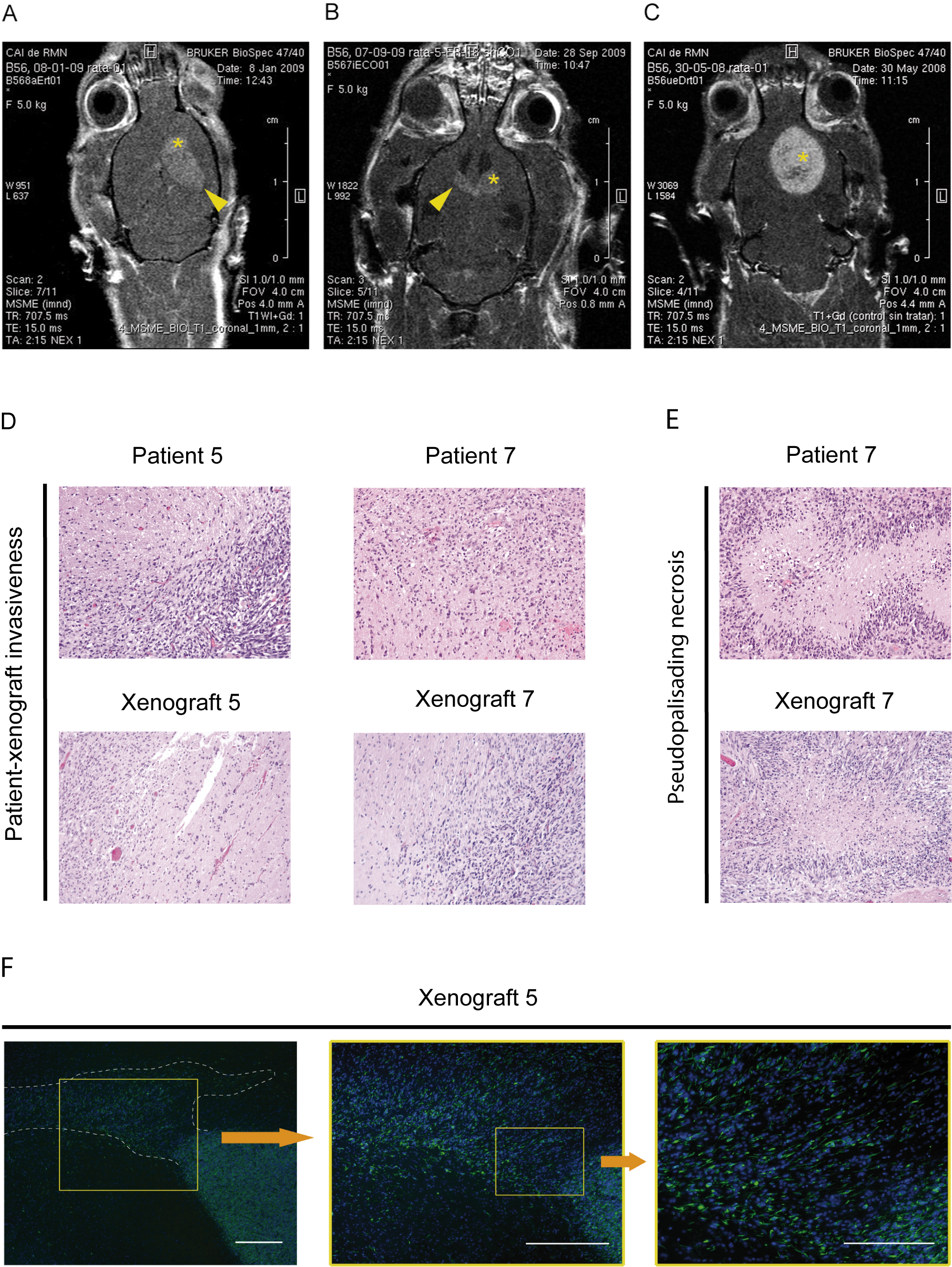

Supplement: Figure S2 — Invasiveness of tumors. A. Magnetic resonance imaging showing the infiltrative capacity of the CSCs-5-induced tumors. Asterisks: inoculation points; arrowheads: tumoral tissue limit. B. Contralateral hemisphere invasion of CSCs-5 cells. C. As a contrast, a much better delimited and less invasive tumor induced by U87MG cells. D. GBM invasiveness confirmation by histology in both patient-xenograft pairs. Hematoxylin and eosin staining. 10× objective. E. Pathologic study showing the pseudopalisading formation and necrosis detail (whiter areas) in patient and xenograft-7. 10× objective. F. Specific anti-human nestin staining (green) in xenograft 5 showing GBM cells infiltrating the cerebral parenchyma. Scale bar indicates 500 µm in the left and center panels, and 250 µm in the right panel. (TIF) [file pone.0028753.s002.tif]

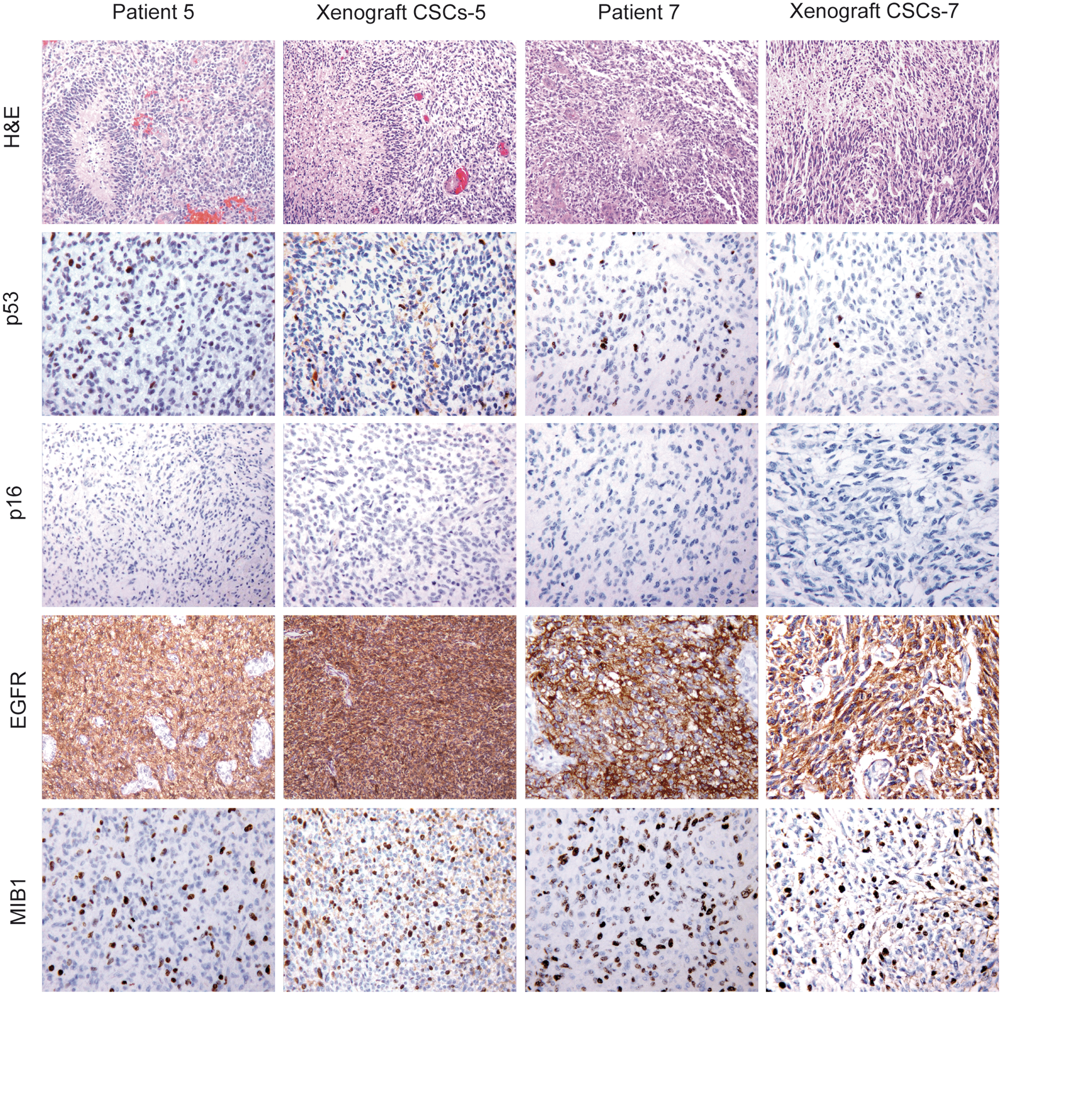

Supplement: Figure S3 — Histopathological analysis of both patients and xenografts tumors. Comparison between xenografts of both patient-derived CSCs and the original tumors of haematoxilin-eosin staining, p53, p16 and EGFR expression, and the proliferative index (MIB-1). (TIF) [file pone.0028753.s003.tif]

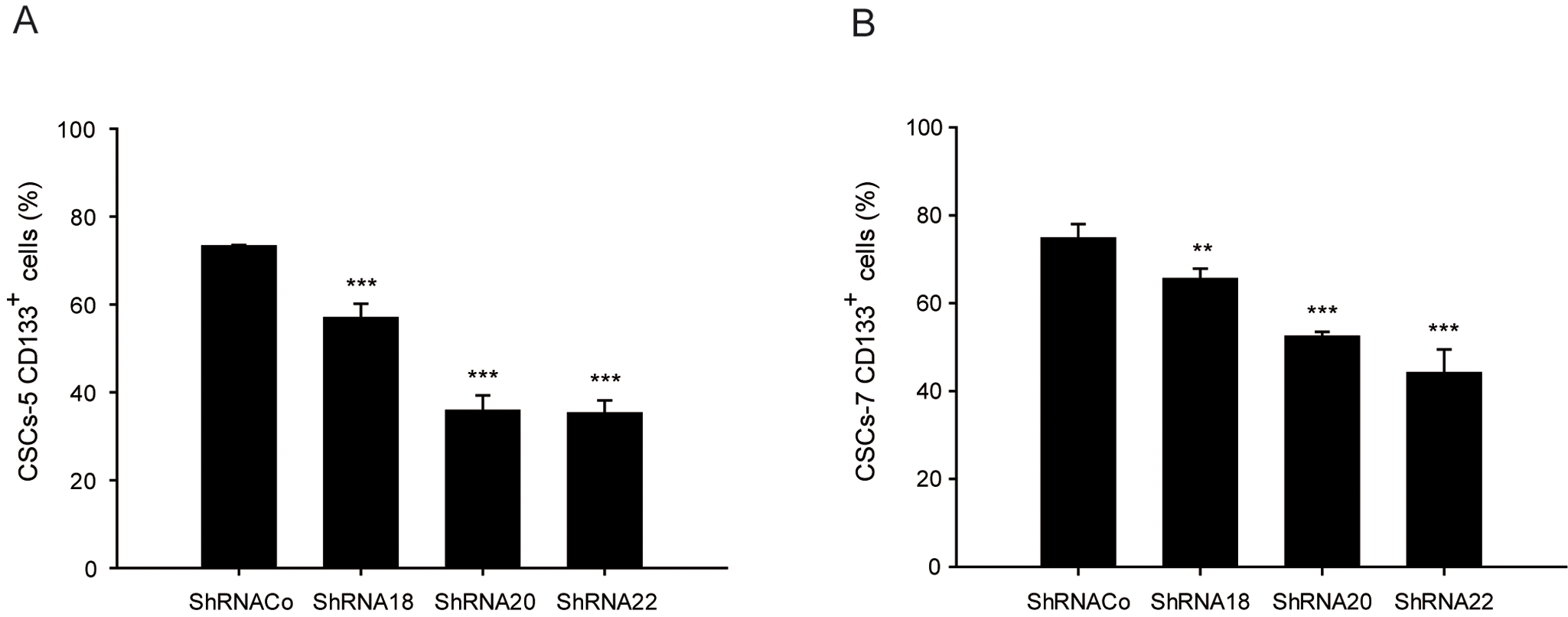

Supplement: Figure S4 — Death of CD133+ cells. A. Percentage of CD133+ cells in CSCs-5 and CSCs-7 (B) cells treated with the different shRNAs. (TIF) [file pone.0028753.s004.tif]
