# Supplementary material for: An Off-Target Nucleostemin RNAi Inhibits Growth in Human Glioblastoma-Derived Cancer Stem Cells
Source: PLoS One. 2011 Dec 12;6(12):e28753. doi: 10.1371/journal.pone.0028753 (PMC3236221; doi:10.1371/journal.pone.0028753)
Supplement: Table S1 — Names of the down-regulated genes when cells are expressing shRNA22, ratio shRNA22/shRNACo, p-value t. test, gene symbol and mRNA accession-number. (DOCX) [file pone.0028753.s005.docx]

Supporting information legend for Table S1

Table S1. Names of the down-regulated genes when cells are expressing shRNA22, ratio shRNA22/shRNACo, p-value t. test, gene symbol and mRNA accession-number.
